# Intelligent Physical Exercise Training (IPET) in the offshore wind industry: a feasibility study with an adjusted conceptual model

**DOI:** 10.1186/s40814-022-01106-z

**Published:** 2022-07-23

**Authors:** Anne Skov Oestergaard, Louise Fleng Sandal, Trine Fernando Smidt, Karen Søgaard

**Affiliations:** 1 Unit for Physical Activity and Health in Working Life, Department of Sports Science and Clinical Biomechanics, University of Southern Denmark, Campusvej 55, Odense M, DK-5230 Odense, Denmark; 2 Ørsted A/S, QHSE (Quality, Health, Safety, Environment) Support, Gentofte, Denmark; 3 Department of Clinical Research, University of Southern Denmark, Odense, Denmark

**Keywords:** Wind technician, Workplace exercise, Offshore wind industry, Musculoskeletal pain

## Abstract

**Background:**

Good physical health and capacity is a requirement for offshore wind service technicians (WTs) who have substantial physical work demands and are exposed to numerous health hazards. Workplace physical exercise has shown promise for improving physical health and work ability among various occupational groups. Therefore, we aimed to assess the feasibility and preliminary efficacy of Intelligent Physical Exercise Training (IPET) among WTs in the offshore wind industry.

**Methods:**

A within-subject design was used to assess the feasibility and preliminary efficacy of IPET (one hour/week individualized exercise during working hours). The intervention period was 12 weeks, with the first eight weeks performed on site as supervised or partly supervised exercise during work hours and the last four weeks planned as home-administered exercise after the seasonal offshore service period. Three assessments, T1 (six months prior to intervention start), T2 (start of intervention) and T3 (end of intervention), of physical health and capacity (self-reported and objective measurements) were conducted and the period between T1 and T2 served as a within-subject control period. Primary outcome was feasibility measured as compliance, adherence, adverse events, and participant acceptability. Descriptive statistics were used to present feasibility outcomes. Preliminary efficacy was reported as mean differences with 95% confidence intervals for health and physical capacity outcomes between T1 and T2, between T2 and T3 and between T1 and T3.

**Results:**

All WTs at the included wind farm (n=24, age: 40 years (SD±8)) participated in the study. No serious adverse events were reported. Compliance and adherence of 95 and 80% respectively, were reached in the eight-week supervised part, but were lower when exercise was home-administered (<20%). Acceptability was high for the supervised part, with 83% indicating that the exercise program worked well and 100% that exercise should be implemented as an integrated part of the working structure. Changes in physical capacity and health indicators, such as VO_2_max (ml O_2_/kg/min) at T1 (38.6 (SD±7.2)), T2 (44.1 (SD±9)) and T3 (45.8 (SD±6.5)), may indicate seasonal fluctuations as well as improvements from the intervention.

**Conclusion:**

On-site Intelligent Physical Exercise Training during working hours was feasible and well received among WTs in the offshore wind industry. The proceeding of larger-scale evaluation and implementation is therefore recommended.

**Trial registration:**

ClinicalTrials.gov (Identifier: NCT04995718). Retrospectively registered on August 6, 2021,

## Key messages regarding feasibility

- Workplace exercise has proved efficient within many occupational groups. However, the acceptability and sustainability of implementing such concepts in different workplace settings remain understudied.
- We found high compliance, adherence, and acceptability of a targeted workplace exercise intervention in an offshore work context among wind service technicians.
- Our study highlights opportunities and nuances essential for successful next phase planning of large-scale implementation of workplace exercise in the offshore wind industry.

## Background

The global wind industry is expanding and consequently, wind service technician (WT) is proportionally one of the fastest growing occupational groups within the labor market today [1, 2]. Offshore WTs spend a high proportion of their working time in the offshore environment where they inspect, maintain, and repair wind turbines in operation [3]. Their work structure and the offshore environment present several known risk factors for developing poor health outcomes like musculoskeletal disorders. Examples include remote work conditions, seasonal work, irregular work/leave schedules, long working shifts, and uncertainty in tasks dependent on weather conditions [4–9]. The prevalence of musculoskeletal disorders among WTs has further been associated with the physical work demands offshore, specifically in terms of turbine type on location (size), the duration of work in awkward postures, and manual handling activities [6, 10]. Objectively assessed physical work demands among WTs support the notion of substantial physical requirements. For example, long distance vertical ladder climbing can be highly physically demanding [11–13]. The average physical workload may additionally exceed the occupational limit of ~30% of maximal capacity during regular work activities, especially among WTs with low physical capacity [11, 14]. However, the volume of high intensity activities (>60% of maximal physical capacity) performed during daily work may not be sufficient for improving or maintaining the required levels of physical capacity and health among WTs [13]. Sustaining sufficiently high physical capacity in relation to the physical work demands seems to be a prerequisite for maintaining work ability [15], productivity [16], and preventing sickness absence and premature retirement from the labor market [17]. Therefore, the current body of evidence suggests that a certain level of physical capacity and health is required for ensuring a sustainable working life as a WT.

Physical capacity assessments and minimal physical requirements required for offshore access have been proposed to and adopted by companies in the offshore wind industry [18–20]. However, there are currently no globally agreed standards and limited empirical evidence to guide these processes [21]. The only physical capacity parameter assessed by offshore operators and contractors as of today is aerobic capacity [21, 22]. A minimal requirement for VO_2_max of 35 ml O_2_/kg/min has been implemented in some parts of the industry [20, 21, 23], although other physical capacities may be equally relevant to manage and improve for ensuring optimal productivity and health among workers [24]. As physical capacity requirements focus on ensuring minimal rather than optimal levels of physical capacity among workers, a shifting focus towards strategies for health and capacity optimization may be valuable. Particularly for WTs and offshore wind companies having difficulties meeting the formal standards for physical requirements [19] but also for a generally more sustainable and healthier working life.

Structured workplace exercise programs have proved effective within a variety of occupational groups [25–28]. The aims of such exercise programs should include improving essential physical capacities, e.g., aerobic and strength, for improved physical work performance and health [26]. Improved physical capacity is generally best obtained by engaging in high intensity and well-structured physical training [29]. Higher capacity has the potential of lowering the relative physical work demands, thereby protecting against insufficient recovery and health implications like musculoskeletal disorders [26, 30]. Structured high-intensity strength—and aerobic workplace exercise, has similarly proved effective for treating musculoskeletal disorders [26]. Strength training as a specific intervention is further recommended for preventing and managing work-related musculoskeletal disorders among employees with physical demanding work [25, 27] and has even shown effective at a very limited dose (i.e., a few minutes per day) [31].

Prioritized accordingly, the concept “Intelligent Physical Exercise Training” (IPET) has been developed, tested, and shown efficient for physical capacity optimization, musculoskeletal symptom reduction, and improvements in work performance across different occupational groups [26, 28]. The exercise concept is a semi-standardized framework and developed to prescribe individually tailored exercise for 1 h per week during working hours based on (1) the physical work demands, (2) individual physical capacities, and (3) the individual physical health profile (e.g., musculoskeletal disorders) of the worker. Specific cut-points for prescription of exercise are risk-based and built on population norms and known physical work demands [32].

While workplace exercise programs have shown effective in multiple settings in short-term efficacy trials, special attention should be paid to the development and feasibility to specific target groups and contexts for successful long-term implementation purposes. To our knowledge, no studies have assessed the efficacy or feasibility of implementing on-site supervised—or home-administered physical exercise training in the offshore wind industry.

The overall objective of this study was to assess the feasibility of implementing an adapted version of IPET targeting WT requirements and work conditions in a real workplace setting as well as home-administered. Specifically, we examined the compliance, adherence, adverse events, and participant acceptability of the intervention (“can it work?”). Secondary, we preliminarily evaluated the within-subject development in physical capacity and musculoskeletal health before and during the intervention period (“does it work?”).

## Methods

### Study design

The present study was a one-armed intervention designed to assess the feasibility and preliminary efficacy of IPET [32] among workers in the offshore wind industry, specifically focusing on WTs and their specific physical work demands. The study period included three assessments of self-reported and objectively measured physical health and capacity; T1 (6 months prior to intervention start), T2 (intervention start), and T3 (after 12 weeks; end of intervention) with the period between T1 and T2 serving as a within-group control period (Fig. 3). The intervention period (T2-T3) was 12 weeks between August and November 2020, with the first 8 weeks taking place on site as supervised or partly supervised exercise during paid working hours. The last 4 weeks were planned as home-administered exercise after termination of the offshore service period when WTs were not on shift. Exercise and assessments were offered to all employees at the participating wind farm (offshore WTs, office-based, and warehouse employees), but this paper focuses on the development and feasibility outcomes for WTs only. All communication (e.g., instructions) and events (e.g., presentations and days of assessments) were co-designed and facilitated with WTs and a local health and safety advisor to assure applicability and acceptability. The trial was retrospectively registered at ClinicalTrials.gov (Identifier: NCT04995718). The reporting in this paper follows the Consolidated Standards of Reporting Trials (CONSORT) 2010 extension to randomized controlled pilot and feasibility trials [33], only including items applicable to the current study design in accordance with Lancaster et al. [34].

### Participants

WTs were recruited from a Danish offshore wind farm, utilizing a 1-week rotational work schedule (1 week on shift with 12-h workdays, 1 week off shift) during the summer service period. In a workshop following up on a workplace assessment survey on health and safety-related topics (WPA), the combined employee group at the wind farm identified musculoskeletal disorders and insufficient physical capacity as highly prioritized challenges related to their daily work. Consequently, they contacted the headquarters of the company for support driving an initiative (fall 2019). Based on the specific request, the present study was preliminarily designed and presented to the responsible site management, health and safety representatives, and WTs for endorsement. Following endorsement, the whole group of WTs decided to sign up for participation as a collective. All WTs were considered eligible for participation but would be excluded from physical capacity assessments if they showed blood pressure above 165 mmHg systolic and 105 mmHg diastolic at T1, T2, or T3. Severe current pain, corresponding to ≥6 on a 0–10 numerical rating scale (NRS), would automatically exclude WTs from strength measurements of the affected body part, but not from study participation.

### Intervention mapping

To investigate the practicality and required modifications to the specific wind farm, six ~ 1-h interviews were conducted with the site manager, operations managers (*n*=2), occupational health representatives appointed to supervise exercise sessions (WTs) (*n*=2), and an additional WT (*n*=1). Based on results from the interviews and a 2-day site visit observing the work environment and facilities, the following suggestions were proposed and accepted by the management: (1) Two weekly exercise sessions of 1 h to be conducted at the offshore facility (on site), equaling 1 h of exercise on average per week; (2) supervisors appointed to facilitate the exercise sessions and track compliance, adherence, and adverse events on behalf of all participants; and (3) exercise sessions to take place at the offshore fitness facility (fitness room with equipment) or at an onshore commercial fitness facility located close to the work site if WTs were away from the offshore platform because of poor weather (membership included).

The most emphasized point made by all interviewees was that the operations management needed to prioritize and plan the weekly exercise sessions and these should be supervised to ensure compliance. The appointed supervisors were familiar with fitness training and were trained by the research team in how to instruct and suggest modifications to colleagues when required. Before the COVID-19 lockdown (March 2020), it was planned that all WTs would receive their individually tailored exercise program in combination with individual instruction by the research team. However, because of COVID-19-related travel restrictions, the supervisors took over responsibility for the initial instruction. All WTs were always welcomed to reach out for support to the research team throughout the intervention period. Thorough instruction manuals and exercise catalogues were handed out to all participants and extra equipment (i.e., resistance differentiated elastic bands) were provided to WTs for the home-administered part of the intervention.

### Intervention

**Fig. 1:**
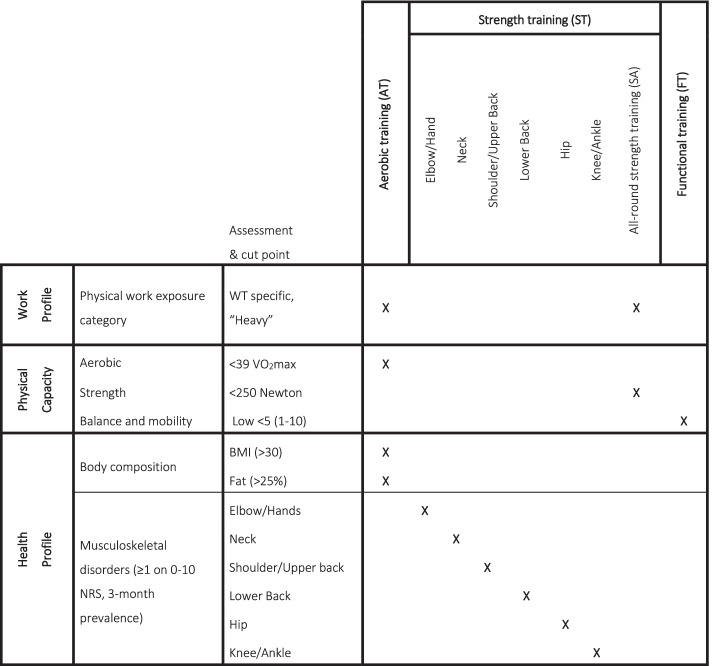
Overview of assessments and cut points used to prescribe individual modes of IPET for WTs. Specific assessments and exercise prescription matrix applied in IPET for WTs. BMI, body mass index; NRS, numerical rating scale

The intervention averaged 1 h of IPET per week for 12 weeks. The exercise prescription was based on sports science principles and physiological theory and individualized according to the physical demand profile, physical capacities, and the physical health profile of the WTs (musculoskeletal disorders and body composition, see Fig. 1). The specific content and rationale of the concept as well as the earlier workplace interventions using it has been described elsewhere [26, 28, 32]. Based on findings from the intervention mapping and development phase of the intervention, the specific contents (e.g., specific exercises and cut points for exercise prescription) were altered to fit the target population of WTs, the offshore work context and available facilities (e.g., fitness room and equipment).

**Fig. 2:**
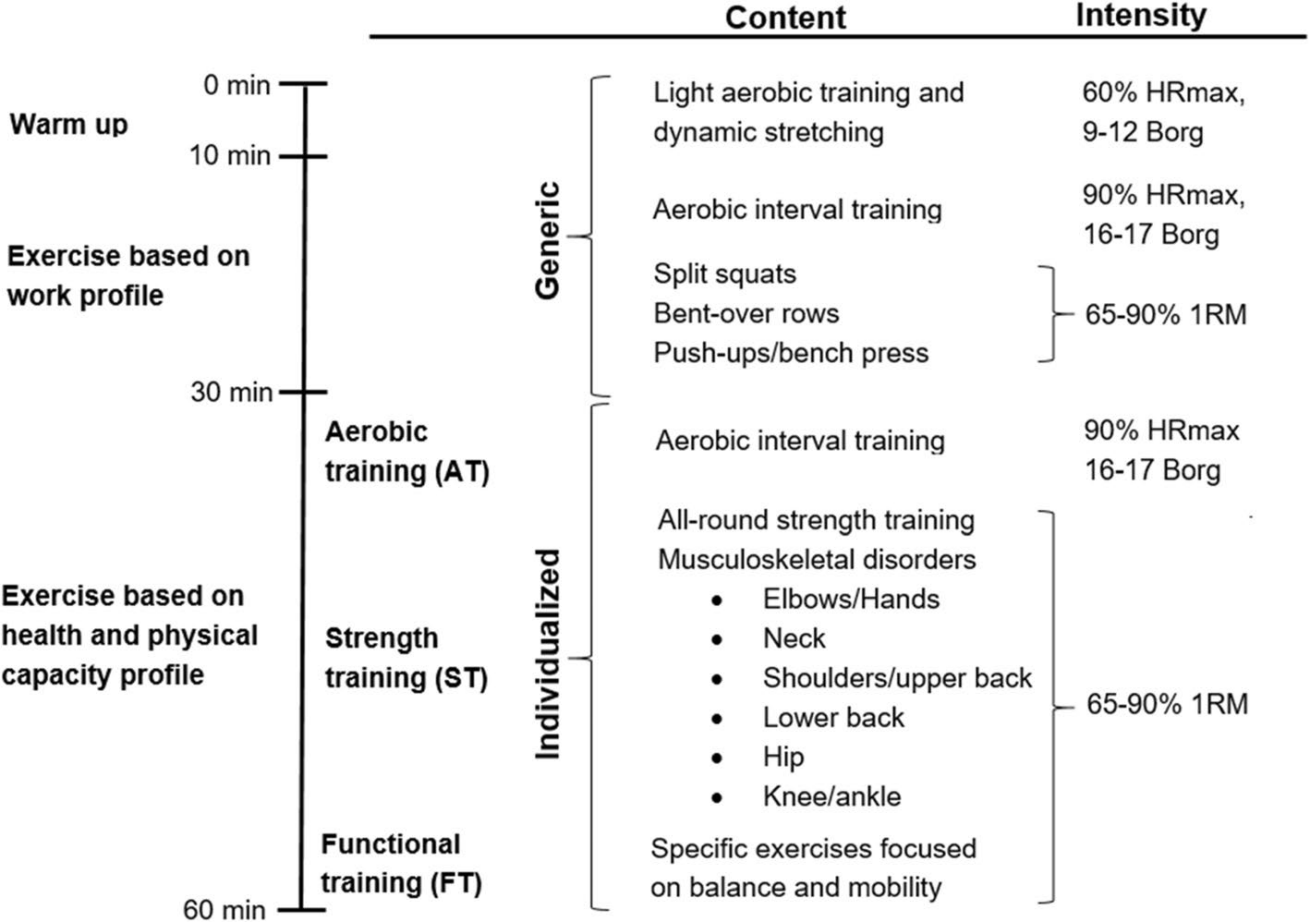
Specific contents, intensities, and durations of the 1-h exercise program. Overview of generic and individualized parts of the exercise prescription. HR_max_, maximal heart rate; Borg, 6–20 point Borg-scale for perceived exertion; RM, repetition maximum (percentage of the maximum weight that can possibly be lifted for one repetition)

The prioritized and included specific cut-points, intensities, and principles used in the modification and development of “IPET for WTs” are specified in Figs. 1 and 2 and in Table 1. Evidence-informed exercise prescription principles published in the American College of Sports Medicine 2009 position stand, which the IPET concept originated from, are further included in Table 1 for comparison.

**Table 1:** Summary of IPET concept and alterations

	ACSM [29]	IPET concept [32]	IPET for WTs	Justification for alterations
Concept	Generic	Individually tailored exercise program informed by occupational demands, musculoskeletal health, and physical performance	Individually tailored exercise program informed by occupational demands, musculoskeletal health, and physical performance	- N/A
Exercises	Compound exercises prioritized	Mix between isolation and compound exercises	Compound exercises prioritized	Compound exercises prioritized based on the physical demand profile (full body, heavy physical) and to allow more sets per muscle group [29]
Equipment	Body weight, elastic bands, and free weights	Body weight, elastic bands, and free weights	Free weights prioritized	Alterations based on availability of equipment and participant preferences (free weights preferred over elastic bands)
No. of set per exercise	6–15 sets per muscle group/week	2–4 sets per muscle group/week (varies)	4–10 sets per muscle group/week (varies)	More sets per muscle group allowed when compound exercises are prioritized over isolation exercises
Intensity	12-8 RM (increasing over time)	15-8 RM (increasing over time)	15-5 RM (increasing over time)	Trained individuals allowed to start at a higher intensity than previously untrained. RIR used to prescribe intensity instead of RM or Borg perceived exertion [35]
Frequency per week	2–3	1 (can be split between up to five shorter sessions)	1 on average (two on offshore rotation weeks, 0 on off-shift weeks)	1-hour sessions preferred by target group and management
Duration per session	~60 min	10–60 min	60 min	- N/A
Time expenditure per week	2–3 h	1 h	1 h	- N/A
Volume	Progressive overload	Steady state	Progressive overload	Increase in volume is considered important for continuous and optimal improvements in physical capacities with exercise over time [29, 36]
Periodization	Linear periodization	Varied (linear, undulating)	Linear	Linear periodization is simple and has shown equally effective among previously untrained individuals [35]
Outcomes	General physical health and capacity	Varied, e.g., musculoskeletal symptoms - Physical capacity - Workplace performance indicators - Relative work intensity	- Feasibility - Musculoskeletal symptoms - Physical capacity - Workplace performance indicators	Based on research aim

*ACSM* American College of Sports Medicine, *IPET* Intelligent Physical Exercise Training, *RM* repetition maximum, *RIR* Reps in Reserve

Figure 1 displays an overview of the tests, questionnaires, and cut points included to prescribe individual modes of exercise in IPET for WTs and is modified from the previous versions published in Sjøgaard et al. [32] and later in Sjøgaard et al. [28]. Briefly, musculoskeletal disorders were evaluated using the Nordic Musculoskeletal Questionnaire (NMQ) where the average 3-month severities ≥1 (0–10 on a numerical rating scale (NRS)) led to prescription of body part specific strength training. If symptoms were perceived in more than two body parts, the prescription of specific strength training was prioritized for the two most affected body parts (or three if additional aerobic, all-round strength training and functional training were not prescribed). The aerobic physical capacity cut-point was based on the company’s current VO_2_max requirement of 35 ml O_2_/kg/min in addition to the standard error of the measurement tool (Chester Step test, 3.9 ml O_2_/kg/min) [22], meaning that aerobic capacities below 39 ml O_2_/kg/min would lead to prescription of aerobic exercise. Strength capacity was measured as isometric shoulder abduction performance using hand-held dynamometry [37] and the cut point was set as the average level obtained within a comparable group of WTs (<250 Newton, data not published). The assessments of balance and mobility were self-reported, and specific exercise was prescribed if participants evaluated their capacities lower than their peers, corresponding to less than five on a 1–10 point (NRS) [38].

Figure 2 shows an overview of the contents, intensities, and durations of the generic and individualized parts of the 1-h exercise program. The generic exercises for the work profile (first 20 min after warm-up) and all-round strength training exercises were proposed based on the identification of physical work demands and musculoskeletal disorder prevalence identified in our previous studies (full-body strength) and finally decided in collaboration with WTs (intervention mapping) [6, 13]. Specifically, strength training exercises for the lower back, neck, shoulders, and knees were prioritized, as well as high-intensity aerobic interval exercise.

### Outcomes

#### Primary outcomes

##### Compliance, adherence, and adverse events

The appointed supervisors tracked compliance, adherence, and potential adverse events during the first 8 weeks of the exercise intervention (192 possible sessions) for all WT participants. Compliance was recorded if a WT attended the planned session. Adherence was reached and reported if the WT completed the exercise program as prescribed, meaning that they did not deviate from the structure of the individual exercise program in terms of exercise selection (or approved alternative exercises), volume, and intensity. Supervisors were asked to record mild (e.g., soreness beyond expected levels) and serious adverse events (safety-related incidents, e.g., dropped objects and sprains/strains associated with conducting the exercises) related to the training sessions. During the last 4 weeks (home-administered exercise), WTs were asked to report attendance in a similar way by phone to the supervisor or health and safety representative or by filling out a record located at the onshore facility at occasion. Missing reports were interpreted as non-compliance and non-adherence.

##### Acceptability

General acceptability and satisfaction with the on-site part of the intervention were evaluated after 8 weeks using a customized survey for the intervention and WTs. Questions concerned: (1) overall acceptability and satisfaction with workplace exercise, (2) the specific content of this intervention, and (3) perceived changes regarding physical capacities and musculoskeletal symptoms. Responses were reported on a 5-point Likert scale (from “Strongly disagree” to Strongly agree”) and using dichotomous Yes/No responses. The acceptability survey further allowed and encouraged WTs to submit open-ended responses elaborating on their expectations and experiences with the intervention. Comments were categorized into main themes after review.

#### Secondary outcomes

##### Objective measures

**Fig. 3:**
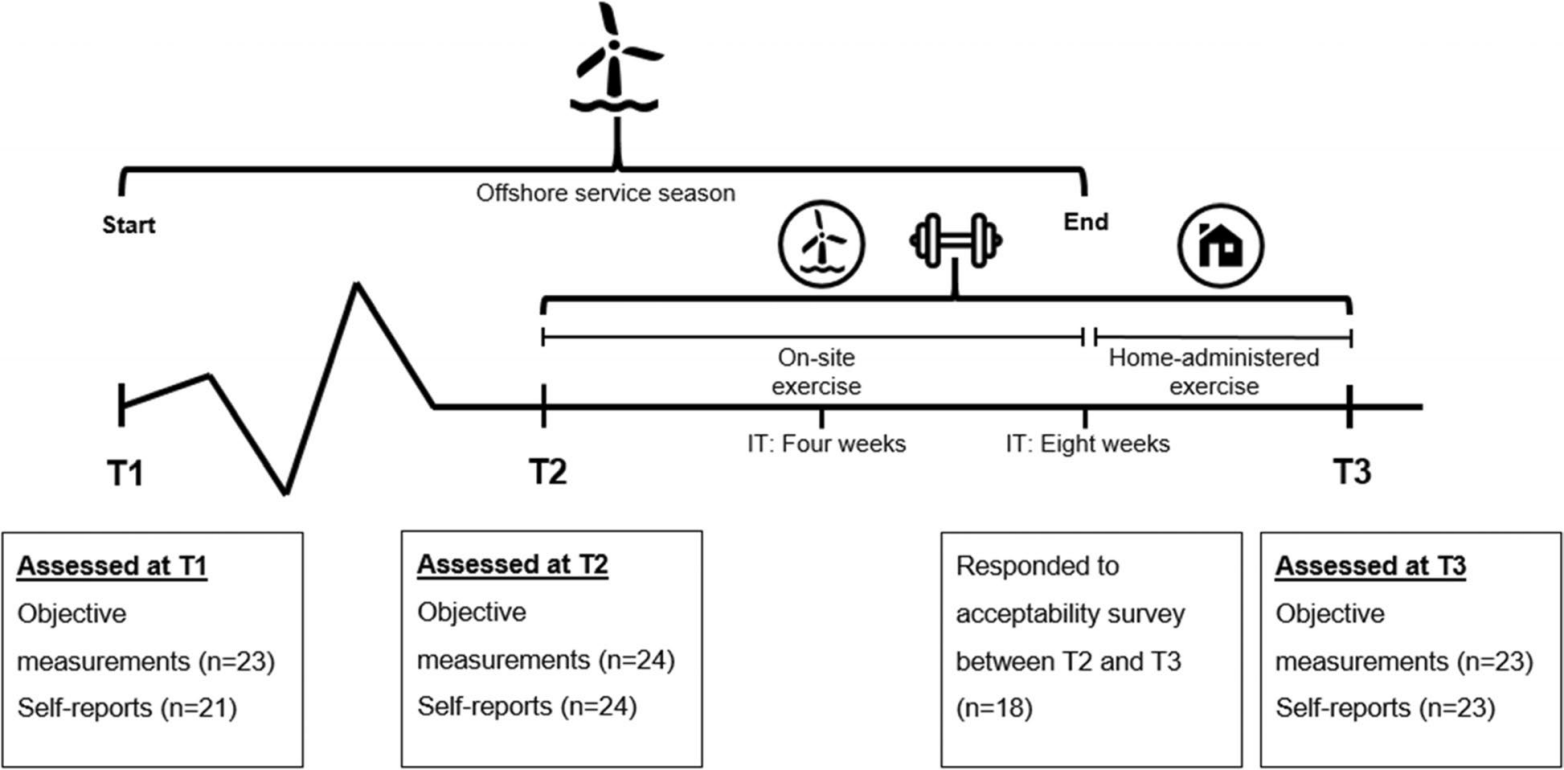
Participant flow and timeline. Notes: The first assessment (T1) of physical health and capacity took place just before the beginning of the offshore service season (end February). The second assessment (T2) was conducted after 6 months (August) and marked the beginning of the intervention (IT) period. The exercise IT period was 12 weeks in total (illustrated with a dumbbell), with the first 8 weeks conducted on-site and the last 4 weeks administered at home as illustrated by the offshore turbine and house pictograms, respectively. The final assessment (T3) marked the end of the intervention and was conducted in November (after 9 months). Acceptability was assessed for the on-site part of the intervention, and survey responses were collected after the first 8 weeks of the intervention

Physical capacity and health checks were conducted at T1, T2, and T3 with the period between T1 and T2 serving as control period and the period between T2 and T3 being the intervention period (see Fig. 3). Some of the physical capacity tests were excluded at T1 because of time restrictions.

The assessment included resting blood pressure, height and waist circumference, and weight and body composition (Tanita TBF-310GS). Aerobic capacity (relative VO_2_max) was indirectly assessed using the submaximal Chester Step Test on a 30-cm step [22] and was estimated using an electronic linear regression of best fit according to identified oxygen equivalents [22]. Subsequently, muscle strength measurements were performed as maximal voluntary contractions (MVC) of the forearm using a hand grip dynamometer [39], isometric shoulder abduction using hand-held dynamometry [37] and a timed side plank (core endurance) [40]. Forearm and shoulder strength were assessed three times for the dominant arm, and the maximal values were used for later reporting. For the side plank, participants were thoroughly instructed to do one maximal duration trial on the preferred side. Finally, back side flexibility was evaluated using the finger-to-floor method [41].

##### Self-reported measures

Workplace performance indicators (work ability and performance [42, 43]) and musculoskeletal disorders [44] were assessed using questionnaires at T1, T2, and T3, and perceived physical capacities [38] were assessed at T1 and T2. Musculoskeletal disorders were assessed in terms of prevalence and severity for nine different body parts and recorded if WTs had experienced symptoms for more than one day and with average symptom severity of ≥ 1 (0–10) within the past 3 months.

### Data protection and ethical approval

The study protocol was registered at the Regional Scientific Ethical Committees for Southern Denmark that stated no ethics approval was required for this study (ID: S-20182000-161). Data protection approval was granted by the University of Southern Denmark (10.286). In accordance with the responsible company’s data policies, written consent was collected from all participating employees and the data was collected and stored pseudonymized. Participants were informed about their right to withdraw individual responses before anonymization of the collected data (no later than five years after project end).

### Statistics

For the primary feasibility outcomes (compliance, adherence, adverse events, and acceptability), categorical distributions were presented as number of WTs and percentage of training sessions/the total population distributed into specific categorical variables. For most other outcomes, we used descriptive statistics, including group means and standard deviations to summarize objective and self-reported measures collected on T1, T2, and T3. For continuous variables, numerical differences and 95% confidence intervals were further used to present differences in outcomes between time points (T1-T2, T2-T3, and T1-T3). All analyses, tables and graphs were carried out using Stata 16, GraphPad Prism version 8.2.1, and Microsoft Word for Microsoft. The sample size was decided by the number of eligible WTs on the recruited wind farm (*n*=24) and within the range of subjects normally recruited for pilot and feasibility studies of this kind [45].

## Results

### Participant flow

All 24 eligible WTs agreed to participate in the exercise intervention and to have their health and physical capacity assessed at the three time points, T1, T2, and T3. All participants further consented to answer questionnaires at the same time points and after the first 8 weeks of the intervention. Missing data occurred on all occasions except at intervention start (T2). Figure 3 displays an overview of the participant flow and timeline.

### Participant characteristics

WTs were male and worked rotational schedules of 7 days on/7 days off work during the offshore service season (March to October). During the offshore work weeks, all WTs had offshore accommodation (living on an offshore accommodation platform) and 12-h working shifts (~6 a.m. to ~6 p.m.). Further baseline characteristics are displayed in Table 2.

**Table 2:** Participant characteristics at baseline (T1)

Variable	Mean (SD)	*N* (percentage)
Age (years)	40 (8)	
Sex (male)		23 (100)
Height (cm)	181 (6)	
Weight (kg)	97 (23)	
Body mass index (kg/m^2^)	28 (4)	
Blood pressure, systolic (mmHg)	149 (14)	
Blood pressure, diastolic (mmHg)	90 (11)	
Smoking (yes)		6 (29)
Living in a relationship (yes)		17 (81)
Seniority in the wind industry (>3 years)		19 (90)
Average perceived exertion during work (6–20)	13 (2)	
Leisure time physical activity level (high)		9 (43)

23 WTs completed the baseline physical capacity and health check and 21 completed the questionnaire at T1. ‘High’ leisure time physical activity level refers to doing light physical activity for more than 4 h per week (on average) or moderate to vigorous physical activity for 2–4 h per week [46]. Perceived exertion during work was assessed on the 6–20 Borg-scale ranging from “No exertion at all” to “Maximal exertion,” with 13 corresponding to “Somewhat hard” [47]

### Feasibility outcomes (compliance, adherence, and adverse events)

Compliance and adherence to the prescribed exercise program were high (>80% on average, range 63–100% and 50–100% for compliance and adherence, respectively) during the first 8 weeks of the intervention (Table 3). Conversely, during the last 4 weeks of the intervention, when exercise was home-administered, only few exercise sessions were attended (13 out of 96) and completed as prescribed (7 out of 96). Although thorough information was given that soreness could be expected in the following days of the initial exercise sessions, three WTs reported soreness as mild adverse events in weeks 1 and 2. No mild or serious adverse events were otherwise reported in connection with the exercise sessions.

**Table 3:** Feasibility outcomes

	On-site supervised exercise sessions (192 possible)	Home-based exercise sessionsaa (96 possible)	Sessions in total (288 possible)
Compliance (attended workout)	183 (95%)	13 (14%)	196 (68%)
Adherence (completed as prescribed)	154 (80%)	7 (7%)	161 (56%)

The appointed supervisors recorded compliance and adherence on behalf of all included WTs (*n*=24) during the on-site supervised part of the intervention. Individual WTs reported for themselves during the home-administered part of the intervention

### Acceptability

Acceptability was rated high as more than 70% of participants found exercises relevant both for specific work requirements and requirements outside of work (Table 4). Eighty-three percent self-reported that one or more physical capacities had improved within the first 8 weeks of the intervention (cardiorespiratory fitness, strength, endurance, flexibility, balance), with the most prevalent being cardiorespiratory fitness (61%) and the least prevalent being balance (11%). Further, 66% indicated that the first 8 weeks improved their musculoskeletal symptoms, 17% reported unchanged symptoms, and 17% that they did not have any symptoms at baseline to improve. Finally, 18 open-ended comments and reflections were received from 14 WTs in connection with the acceptability survey. Identified categories and examples are outlined in Table 5. Generally, they presented within four main themes, and both barriers and opportunities for further implementation of workplace exercise were highlighted.

**Table 4:** Acceptability (participant satisfaction and evaluation)

On-site supervised exercise overall (*N*=18)	Strongly or partly agreed, *N* (%)
The exercise program worked well overall during the summer rotation	15 (83)
Workplace exercise is relevant for requirements outside of work	13 (72)
Workplace exercise is relevant for our work requirements as wind technicians	17 (94)
One hour was sufficient to complete all prescribed elements	12 (67)
The fitness room was large enough and we had the required equipment	11 (61)
It was possible to adjust exercises as needed	15 (83)
Exercise should be implemented as a part of the offshore work structure	18 (100)
**“Which specific elements has worked well”**	**“Yes”,** ***N*** **(%)**
Warm-up	6 (33)
Cardiorespiratory fitness training (high intensity interval training)	15 (83)
Workplace exposure specific training (the exercises that were the same for all)	9 (50)
Individually prescribed exercises for musculoskeletal symptoms	13 (72)
No elements worked well	0 (0)
All elements worked well	10 (56)

**Table 5:** Open-ended comments from acceptability survey

Category	***N*** (percentage)	Examples
Organization, planning, and supervision	5 (28)	“It is great that technicians have individualized exercise programs, but more efforts should be made ensuring that it gets prioritized by our management.” “Being a wind technician is extremely physically demanding, so of course physical training should be a prioritized part of the work structure.”
COVID-19 implications	5 (28)	“I think the exercises have been appropriately tailored for me, but because of COVID-19 it has been difficult to follow the program and get it done in a fitness center (onshore).”
General comments (reflections and satisfaction)	5 (28)	“When I have completed a workout, I feel improved physical well-being. Generally, I have less pain in muscles and joints than before we started.” “I think it is really great to have the opportunity to exercise during working hours and I feel motivated to continue”.
Structure and content of exercise program	3 (17)	“I would have benefitted more from a team-based exercise structure, since I am not good at exercising on my own” “There should be an alternative, like going for a walk e.g..”

*N*= 14 unique participants leaving 18 comments

### Preliminary efficacy

The physical health and capacity assessments at T1, T2, and T3 revealed changes over time as indicated by 95% confidence intervals of the differences between timepoints, both during the control period and the intervention period. Specific changes over time for the objective measurements are displayed in Table 6.

**Table 6:** Within-subject changes over time (objective measures, T1, T2, T3)

Variable	Mean (SD)			Mean difference [95%CI]		
	T1	T2	T3	T1-T2 Δ	T2-T3 Δ	T1-T3 Δ
BMI (kg/m^2^)	28.1 (4.2)	28 (4.1)	27.5 (3.9)	−0.1 [0.2 to −0.4]	−0.5 [−0.8 to −0.1]	−0.6 [−1 to −0.2]
Systolic BP (mmHg)	148 (14)	140 (14)	138 (12)	−8 [−16 to −1]	−2 [−5 to 1]	−10 [−17 to −3]
Diastolic BP (mmHg)	89 (11)	90 (10)	91 (9)	1 [−2 to 5]	1 [−2 to 3]	2 [−2 to 6]
Fat percentage (%)	23.7 (6.1)	23 (5.9)	22.4 (5.8)	−0.6 [0.2 to −1.4]	−0.7 [−1.2 to −0.1]	−1.3 [−2.3 to −0.4]
Muscle mass (kg)	65.9 (7.1)	66.5 (7)	65.9 (6.5)	0.6 [0.8 to 1.2]	−0.7 [−1.2 to −0.1]	0 [−0.7 to 0.7]
Waist/hip ratio	1 (0.06)	0.96 (0.07)	0.96 (0.07)	−0.04 [−0.07 to −0.01]	0 [−0.03 to 0.03]	−0.04 [−0.06 to −0.01]
VO_2_max (ml O_2_/kg/min)	38.6 (7.2)	44.1 (9)	45.8 (6.5)	5.4 [1.7 to 9.1]	1.7 [−1.4 to 4.7]	7.1 [4.6 to 9.6]
Handgrip strength (kg)	60 (8)	-	61.2 (7.7)	-	-	1.2 [−1.9 to 4.2]
Shoulder strength (kg)	-	29 (4.4)	29.6 (3.9)	-	0.6 [−0.7 to 1.9]	-
Side plank (s)	-	83 (30)	87 (31)	-	4.4 [−3.4 to 12.5]	-
Finger-to-floor (cm)	-	+ 1.2 (8.5)	−0.4 (7.8)		−1.7 [−3.7 to 0.5]	-

*N*=22. Two WTs were excluded from the analysis because they did not have measurements from all time points (T1, T2, and T3). Some tests were not included in the T1 test battery because of limited time to conduct the tests. Handgrip strength is missing at T2 as the dynamometer failed. *BP* blood pressure

The development in musculoskeletal disorders, self-reported physical capacities, and work performance indicators are further outlined in Table 7.

**Table 7:** Within-subject changes over time (self-reported measures T1, T2, T3)

Variable	Mean (SD)			Difference [95% CI]		
	T1	T2	T3	T1-T2 Δ	T2-T3 Δ	T1-T3 Δ
Musculoskeletal disorders (number)	3.1 (2.1)	3.7 (1.7)	2.8 (1.7)	0.6 [−0.2 to 1.3]	−0.9 [−0.1 to −1.6]	−0.3 [−1.2 to 0.6]
Musculoskeletal disorders (severity)	3.6 (2.3)	3.7 (2.1)	3.8 (2.1)	0.1 [−0.6 to 0.9]	0.1 [−0.6 to 0.9]	0.2 [−0.6 to 1]
Work ability (0−10)	8.9 (0.8)	8.7 (0.9)	8.9 (1.1)	−0.2 [−0.6 to 0.2]	0.2 [−0.2 to 0.6]	0 [−0.5 to 0.5]
Productivity (0−10)	8.4 (1.5)	8.5 (0.9)	-	0.1 [−0.6 to 0.5]	-	-
Physical capacities (1−10)						
Cardiorespiratory fitness	5.5 (1.8)	5.7 (1.9)	-	0.2 [−0.4 to 0.9]	-	-
Muscle strength	6.4 (1.8)	6.6 (1.5)	-	0.2 [−0.4 to 0.8]	-	-
Endurance	6.3 (2.1)	6.7 (1.5)	-	0.3 [−0.2 to 1]	-	-
Flexibility	5.7 (2.2)	6.1 (1.9)	-	0.4 [−0.3 to 1.2]	-	-
Balance	6.3 (2.1)	6.6 (1.9)	-	0.3 [−0.3 to 0.9]	-	-

*N*= 20. All WTs responded to a minimum of two out of three surveys. Four WTs had one timepoint without responses and were excluded from the analysis. The survey at T3 only included questions on musculoskeletal disorders and work ability. Musculoskeletal disorders (number) refer to 3-month prevalence (3-month recall period) of any symptoms in up to nine body parts (present > 0 days and with an average severity of ≥ 1 (*NRS* = numerical rating scale 0–10). Musculoskeletal disorders (severity) reflect the average severity (0–10) of all cases combined

## Discussion

### Summary of findings

Feasibility of the intervention was measured with outcomes related to compliance, adherence, adverse events, and acceptability among the target population of WTs in this study. Findings included no serious adverse events, and high compliance and adherence to the intervention in the supervised part (first 8 weeks) but not when exercise was home-administered after the end of the offshore service season. Acceptability was generally high, with 83% of WTs indicating that the specific exercise program worked well during the offshore summer rotation and 100% that exercise should be implemented as a fixed part of the working structure. Objective and self-reported measures collected at T1, T2, and T3 showed potential effects of the intervention on physical capacity and health, but interestingly also seasonal fluctuations, reflected as differences in parameters between timepoints (including the control period).

### Feasibility

Generally, high compliance (95% attendance) and adherence (80% of sessions completed as prescribed) were reached during the supervised part of the intervention which took place at the offshore accommodation platform or at the local onshore fitness center on poor weather days. The high compliance was not surprising since everyone (management included, *n*=4) collectively committed to plan, support, and trial the exercise intervention. Therefore, participation was mandatory for all WTs, even though it was possible not to consent to be included in the study.

Similar IPET trials have mostly offered other types of arrangement for exercise at the workplace [26, 28], and simple comparison of compliance and adherence levels are therefore not directly applicable between studies. Lower compliance and/or adherence levels have, however, been observed when similar populations (i.e., predominantly male occupational groups with high physical work demands) were offered tailored exercise within the IPET framework [48, 49]. For example, the overall attendance rate was 68% among construction workers during a similar 12-week exercise intervention at the worksite [48]. The attendance rate of 95% (compliance) among WTs must therefore be considered high—and a strength of the approach, albeit the differences in utilized designs and inclusion procedures.

Although most of the sessions were attended, a few open-ended comments (*n*=3) from the acceptability survey indicated that high attendance would not be possible without management support and facilitation, e.g., “It is great that technicians have individualized exercise programs, but more efforts should be made ensuring that it gets prioritized by our management”. The low compliance and adherence during the home-administered part of the intervention support that management commitment, and a framework where exercise is supervised and potentially mandatory, are important factors for ensuring successful and sustainable implementation of workplace exercise interventions [50, 51].

Some of the attended sessions (15% during the supervised part) were further not completed as prescribed, which could be due to high complexity of the individual exercise plans, lack of motivation, interest, or perceived relevance with the specific exercise regime, all barriers identified in connection with earlier exercise interventions at the workplace [52, 53]. Until now, no feasibility trials including participants’ satisfaction and evaluation with the IPET exercise concept have been conducted. The present results for feasibility may therefore help guide improvements prior to larger-scale trials and implementation, e.g., related to specific content and participant preferences, and therefore ensure sustained high satisfaction with the specific structure and contents of the program.

### Generic versus individualized exercise prescription at the workplace?

Results from the acceptability survey showed a high level of satisfaction with most of the specific elements and exercises included in the IPET program. It seemed, however, that some elements, specifically warm-up and generic exercises targeted to the work profile, scored a bit lower (Table 4). Common for these elements was that they were generic (although targeted the overall group and work demands) and therefore prescribed equally for all participating WTs. The generally higher satisfaction with the individualized elements of the exercise program may indicate that WTs predominantly prefer an individualized approach to exercise prescription. Previous research shows that individualizing exercise prescription and including participant preferences increase attendance and improves motivation compared with using generic and standardized approaches [54]. Further, individualized exercise increases outcome effect sizes and minimizes adverse events compared with generic exercise prescription [55, 56]. Therefore, individualized exercise prescription should be aimed for when implementing exercise at the workplace for ensuring sustained high adherence and participation rates as well as optimal physical health and capacity outcomes [53].

Even though WTs present as a relatively homogeneous population currently (predominantly male, young, and healthy population) [5, 6], results from this study show that their preferences for exercise prescription are highly individual. For example, one WTs expressed that he “ … would have benefitted more from a team-based exercise structure, since I am not good at exercising on my own” and another one that “there should be an alternative, like going for a walk”. Although preferences varied across the sample, our results showed that 83% of WTs felt they could adjust the prescribed exercises to fit their individual needs within the framework. While the IPET concept is only semi-standardized and individualized to target both individual—and workplace needs, the scope of the concept could potentially be enlarged to allow for even more individualization and participant preference [28]. As a modification from the original IPET concept, this present trial allowed some deviation from the primary prescribed exercises within a wider structured exercise framework, e.g., to utilize alternative equipment where applicable and different exercises, all targeting the same muscle groups. Modification of intensity or primary muscle groups targeted was not permitted, unless severe pain or other implications restricted WTs in following the protocol. Generally, sufficiently high exercise intensity is known to be essential for ensuring optimal results (as high as possible effect sizes for physical capacity outcomes), especially when time for exercise is limited [36, 57, 58]. Therefore, exercise intensity should be one of the most important parameters for employers when deciding if supporting workplace exercise offers the required benefits from a company perspective (e.g., economical). Consequently, standardization of some parameters, like exercise intensity, seem to be essential for effective, viable, large-scale, and long-term implementation since most companies do not have dedicated resources or competences available to individualize or 1:1 supervise workplace exercise programs [32].

### Seasonal fluctuations in physical capacity and health

Unintentionally, we observed a general increase in aerobic capacity and other health—and physical capacity indicators over the full course of the offshore service period (T1-T3) as indicated by 95% confidence intervals in Table 6. The physical work exposures vary between onshore and offshore work among WTs [13] and is generally expected to fluctuate over the year, with the highest physical exposure during the offshore service (summer) period. Preliminary results from this present study indicate that the expected higher occupational physical activity and demands are likely affecting the physical capacity—and health profile positively going from the winter off-season (T1) to summer on-season (T2). However, it is similarly likely that the peak bodily strain during the offshore service season could result in increased bodily discomfort over the course of the offshore season. The relative ~20% increase in musculoskeletal disorder prevalence from T1 to T2 may support such an association (Table 6).

These possible seasonal variations in exposure, physical capacity, and musculoskeletal health among WTs could be relevant for further targeted concept development and future planning of physical capacity maintenance and optimization programs. With seasonal fluctuations in physical demands, workload, stress levels, and musculoskeletal disorders [7], seasonal workers like WTs may benefit from an altered periodization schedule than the linear approach assessed with the IPET concept until now [26]. Like within competitive sport and physical performance optimization, where supportive strength and conditioning programs are usually planned to support peak physical capacity and injury prevention during specific competitive seasons [59], similar approaches may be adopted by seasonal work industries like offshore wind. Accordingly, workplace exercise could be initiated before season start (for example 1 month prior), and vary in terms of volume, intensity, frequency etc., throughout the year to better fit the offshore service season, peak exposures, and required physical performance [59]. These insights may offer practical implications and opportunities for companies’ planning of physical capacity tests and physical exercise implementation at the workplace. Nevertheless, the emerged hypothesis that workplace exercise can minimize seasonal fluctuations in physical capacity, and help ensure health and safety of workers, seems plausible and should be investigated further.

### Strengths and limitations

The primary strength of this study was that many elements of the intervention were feasibility tested and that a pragmatic approach was adopted to allow participant and management involvement in the design. Generally, the application of feasibility studies has the potential to reduce trial costs of large-scale randomized controlled trials and implementation, which can increase the likelihood of success of interventions [60]. It was therefore relevant to determine if the intervention showed promise of being successful with the intended population and if preliminary efficacy could be shown [34]. A particular disadvantage of the one-armed design used in this study was the potential for confounding due to time-related and environmental effects, which may have showed as seasonal fluctuations in physical capacity and health indicators (Table 6). Other limitations include COVID-19-related factors. First, the study was postponed due to travel and exercise restrictions (i.e., fitness facility lock-down), which prolonged the control period between T1 and T2 and shortened the on-site intervention period from 12 weeks to 8 weeks. Second, it was not possible for the exercise physiologist to introduce and instruct the exercise program individually on-site, which added larger responsibility to WT supervisors and limited assurance for a sufficient and standardized approach. However, larger initial supervisor responsibility may have also presented a strength and increased the potential for sustainability through increased WT involvement and accountability. Finally, comments from the acceptability survey and discussions with management implied that derived effects of the pandemic may have included lack of motivation to exercise by WTs and other organizational priorities, potentially resulting in lower managerial support and instructor fidelity than what would otherwise have been expected.

## Conclusion and practical implications

The present results indicate that 1 h per week of structured exercise performed during working hours is feasible and well accepted by offshore WTs and that the IPET intervention may therefore be an effective tool for increasing physical capacity and improving musculoskeletal health in the offshore work setting. The results of the present study therefore justify the proceeding to larger-scale effectiveness and implementation trials.

As the offshore wind industry is expanding and diversifying in the current years, the positive results from this specific wind farm may not be directly translatable to other wind farms, national, or cultural settings across the industry; however, individual site management and WTs should continuously be involved in iterating the concept to ensure optimal implementation and sustainability going forward.

## Data Availability

The datasets generated as a part of the current study are not publicly available but can be requested from the corresponding author on reasonable request (anonymized).
